# Safety and efficacy of P2Et extract from *Caesalpinia spinosa* in breast cancer patients: study protocol for a randomized double blind phase II clinical trial (CS003-BC)

**DOI:** 10.1186/s12906-023-04139-w

**Published:** 2023-09-05

**Authors:** Ricardo Ballesteros-Ramírez, Paola Pinilla, Jesús Sanchéz, Lilian Torregrosa, Pablo Aschner, Claudia Urueña, Susana Fiorentino

**Affiliations:** 1 Grupo de Inmunobiología Y Biología Celular, Facultad de Ciencias, Pontificia Universidad Javeriana, Carrera 7 No. 43-82, Edificio Félix Restrepo, Lab 101., C.P.110211 Bogotá, Colombia; 2 Centro Javeriano de Oncología, Hospital Universitario San Ignacio, Bogotá, Colombia; 3 Oficina de Investigaciones, Hospital Universitario San Ignacio, Bogotá, Colombia

**Keywords:** *Caesalpinia spinose*, Herbal Drugs, Safety, Efficacy, Immunomodulation, Breast Cancer

## Abstract

**Background:**

Chemotherapy in breast cancer is effective but can generate significant toxicity and lead to tumor resistance. Joint treatment with standardized plant extracts can be an alternative to improve the response and allow an effective activation of the antitumor immune response that favors recovery in the short and long term. The P2Et extract of Caesalpinia spinosa presents antitumor activity in cells and animal models of breast cancer, improves the tumor microenvironment, and induces activation of the specific immune response against the tumor and is synergistic when used together with anthracyclines, which makes it a good candidate for evaluation in patients.

**Methods:**

Conducted at a single center, this phase II study is a randomized, double-blind, placebo-controlled trial aimed at assessing the safety and efficacy of P2Et extract in patients diagnosed with stage II and III breast cancer, who are eligible for neoadjuvant treatment. The study aims to determine the safety profile at the previously established optimal biological dose from phase I trial while investigating various efficacy outcomes. These outcomes include improvements in quality of life, immunomodulation, metabolic profile, microbiome, as well as clinical indicators such as tumor reduction, disease-free survival, and pathological response, assessed at different stages of the treatment regimen.

**Discussion:**

Treatment with the P2Et extract in breast cancer patients is hypothesized to enhance overall well-being, positively influencing their quality of life, while also triggering an antitumor immune response and enhancing immune infiltration. These combined effects have the potential to contribute to improved long-term survival outcomes for patients receiving the phytomedicine alongside neoadjuvant chemotherapy treatment.

**Trial registration:**

This trial was registered in the US National Library of Medicine with identifier NCT05007444. First Registered August 16^th^, 2021. Last Updated: August 9^th^, 2022.

**Supplementary Information:**

The online version contains supplementary material available at 10.1186/s12906-023-04139-w.

## Introduction

### Background and rationale {6a}

Breast cancer stands as the most prevalent malignancy among women worldwide, with a staggering 2 million new cases reported in 2020 (11.7% of all cancers) and 684,996 recorded deaths (6.9%) [1]. In Colombia, the disease exhibits an incidence rate of 13.7%, accounting for 15,509 newly diagnosed cases. The 5-year prevalence is estimated at 52,025 cases, boasting an impressive survival rate of over 85% [2]. In addition to clinical factors and tumor biology, the presence of an inflammatory microenvironment can significantly impact cancer initiation and progression [3–5]. Notably, in breast cancer, a heightened immune infiltrate characterized by a diverse T lymphocyte (LT) response has been associated with improved outcomes [6], particularly in HER2 negative patients where CD8 LT infiltration has shown favorable results [7, 8]. Furthermore, increased immune infiltration has been linked to enhanced response rates to neoadjuvant therapy and chemotherapy [9]. This association is accompanied by a reduction in tumor proliferation, as measured by a decrease in intratumoral Ki67 expression [10, 11]. These findings underscore the significance of immune infiltration in breast cancer, as it not only influences prognosis but also affects the response to therapeutic interventions.

In breast cancer, the tumor microenvironment presents a particularly intriguing opportunity for the incorporation of immunogenicity inducers in both neoadjuvant and adjuvant therapies. Primary tumors, in comparison to metastases, have been found to exhibit higher immunogenicity, making them an attractive target for intervention [12]. Bioactive chemicals are abundant in natural goods and traditional herbal medicine, but few herbal formulations have been subjected to scientific testing and confirmation for their potential as medical treatments [13]. However, phytotherapy holds promise as a burgeoning field in contemporary medicine. Derived from plants, phytotherapeutic compounds possess immunomodulatory properties and demonstrate the ability to combat tumors. They foster the activation of immune responses and contribute to reversing the tumor phenotype by acting on the tumor microenvironment [14].

Over the past 15 years, the standardized extract of *Caesalpinia spinosa*, known as P2Et and investigated in this protocol, has exhibited remarkable antioxidant activity both intracellularly and in vitro. Its antioxidant potential surpasses that of commonly used positive controls like TROLOX. P2Et has also demonstrated the ability to modulate drug resistance pumps, thereby enhancing sensitivity to anthracyclines in both in vitro and in vivo settings [15]. Furthermore, P2Et has been found to induce apoptosis by depolarizing mitochondria in various tumor cells, specifically displaying this effect in murine breast cancer cells. This mechanism of action leads to a reduction in clonogenicity and has been associated with diminished primary tumor growth and metastases in animal models of breast cancer. Notably, P2Et has also been observed to decrease serum IL-6 production in these animal models [16]. The tumor cell death process triggered by P2Et is accompanied by the expression of immunogenic death markers, including calreticulin, HMGB1, and ATP secretion which play a crucial role in activating dendritic cells, facilitating the recruitment and differentiation of tumor-specific T lymphocytes (TL) in animal models. In fact, vaccination with tumor cells treated with P2Et leads to the activation of TL in draining lymph nodes, resulting in cytokine production in response to tumor antigens. Notably, the tumor-specific immune response induced by P2Et appears to surpass that of Doxorubicin, as TL generated in response to P2Et exhibit greater multifunctionality in terms of cytokine production [17]. In addition, P2Et can revert protumorigenic fibroblasts of the CAF phenotype to normal cells, decreasing the tumorigenic signals provided by the tumor microenvironment, which can reverse the tumor process [18].

Based on the preclinical safety regulatory assessments, it has been established that P2Et is safe for oral consumption and does not exhibit toxicity [19], mutagenic, or genotoxic properties [20]. Additionally, in the phase I clinical study conducted on healthy volunteers and in the phase II COVID19 patients the P2Et demonstrated a good safety profile [21]. Given this background, it is hypothesized that treatment with the P2Et extract in patients with breast cancer can have a positive impact on their overall well-being, influencing their quality of life. Furthermore, it is believed that P2Et has the potential to induce an antitumor immune response, leading to improvements in immune infiltrate and transforming a "cold" tumor into a "hot" tumor. Such transformations have been associated with enhanced therapeutic outcomes. Consequently, the administration of P2Et in conjunction with the chemotherapeutic treatment selected by the treating oncologist may result in improved long-term survival rates and improve the quality of life for breast cancer patients. To progress further along this trajectory, this phase II study aims to evaluate the safety of P2Et and explore its efficacy outcomes in patients with breast cancer who are undergoing neoadjuvant chemotherapy.

### Objectives {7}

The primary objective of this study is to evaluate the safety and efficacy of P2Et extract in improving the quality of life for breast cancer patients, as well as to investigate various treatment outcomes when combined with chemotherapy. These outcomes encompass the impact of P2Et extract and neoadjuvant chemotherapy on both clinical and pathological response, disease-free survival, and overall quality of life in patients with breast cancer. Additionally, the safety profile of the P2Et extract in conjunction with neoadjuvant chemotherapy will be assessed. The QLQ C-30 version 3 and QLQ B23 questionnaires will be utilized to comprehensively evaluate the patients' quality of life. Moreover, as part of exploratory objectives, several additional aspects of treatment will be examined including the assessment of intratumoral immune infiltrate, tumor proliferation, and the presence of tumor stem cells before, during, and after treatment. Furthermore, the immune response in peripheral blood will be analyzed at different stages of treatment. The patients' plasma metabolome will also be assessed throughout the treatment course to identify potential treatment-related metabolic changes. Lastly, an analysis of the patients' microbiota will be conducted before and after treatment to explore any potential influence on treatment response.

## Methods: participants, interventions, and outcomes

### Trial design and setting {8, 9}

**Fig. 1:**
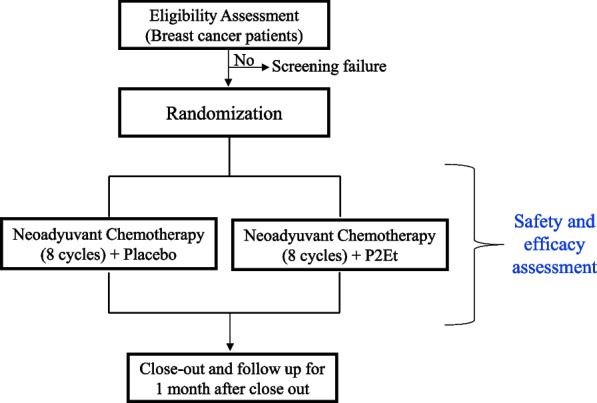
Comprehensive flow diagram for phase II (CS003-BC) in breast cancer patients

This single-center phase II study employs a randomized, double-blind, placebo-controlled design to assess the safety and efficacy of the P2Et. Upon enrollment, patients will be randomly assigned to either the intervention group or the placebo group. Throughout their neoadjuvant treatment and prior to surgery, patients in the intervention group will receive standard chemotherapy along with the P2Et, while those in the placebo group will receive standard chemotherapy alongside a placebo (Fig. 1).

### Eligibility criteria {10}

#### Inclusion Criteria

1. Adult women aged 18 years or older.
2. Patients diagnosed with invasive breast cancer stages II-III who are candidates for standard neoadjuvant chemotherapy.
3. Documented results of estrogen receptor (ER), progesterone receptor (PR), KI-67, and human epidermal growth factor receptor 2 (HER2) status.
4. Patients eligible for neoadjuvant treatment with AC (Doxorubicin/cyclophosphamide) every 2 or 3 weeks followed by taxanes (paclitaxel for 12 weeks or docetaxel every 3 weeks for 4 cycles).
5. ECOG performance status of 0 to 1 with a life expectancy greater than 3 months.
6. The subject is capable of swallowing and retaining oral medication and does not have uncontrolled vomiting or persistent diarrhea.
7. Adequate renal, hematological, and hepatic function as determined by the investigator's discretion.
8. No significant or uncontrolled comorbidities determined by medical history, physical examination, and screening laboratories at the investigator's discretion. (White blood cells > 2000/mm3, neutrophils > 1500/mm3, hemoglobin > 9 g/dL, creatinine < 1.5 times the upper limit, transaminases < 3 times the upper limit, bilirubin < 1.5 times the upper limit).
9. Premenopausal patients without reliable non-hormonal contraceptive methods must have a negative pregnancy test before screening and before each treatment cycle.
10. Female fertile subjects (those not in menopause for at least 12 months or surgically sterile through bilateral tubal ligation, bilateral oophorectomy, or hysterectomy) and their male partners must use at least one of the following contraceptive methods during study entry, throughout the study, and for at least 6 months after discontinuation of P2Et extract use (the effects of P2Et extract on the developing human fetus are unknown):
  Total abstinence from sexual intercourse, starting at least one complete menstrual cycle before administration of the study drug (note: sexual abstinence as a contraceptive method should be limited to cases where it is already established as the patient's pre-existing lifestyle choice).
  Vasectomy in the female subject's partner.
  Intrauterine device (IUD)
  Double barrier method (condom, contraceptive sponge, diaphragm, or vaginal ring with spermicidal jelly or cream).
11. Willingness to comply with study interventions and follow-up.

#### Exclusion criteria

Patients with one or more of the following conditions are not eligible for this study:

1. Patients treated in any other therapeutic clinical protocol within 30 days prior to study entry or during participation in the study.
2. Patients currently receiving other investigational agents.
3. History of allergic reactions attributed to polyphenol compounds similar to those found in green tea.
4. The subject is pregnant or breastfeeding.
5. Concurrent severe active morbidity at the investigator's discretion.
6. Subjects with malabsorption syndrome or any condition affecting the enteral route of administration.
7. Subjects with a confirmed diagnosis of HIV prior to enrollment or a positive HIV diagnosis at the screening.
8. Patients who have received solid organ or hematopoietic component transplantation.
9. The use of other phytomedicines, vitamins, or herbal supplements should have been discontinued at least one week before study entry.
10. Active previous malignancy within the past 3 years, except for locally curable cancers that have apparently been cured, such as basal or squamous cell skin cancer, superficial bladder cancer, or in situ carcinoma of the prostate, cervix, or breast.
11. Any condition that, at the discretion of the principal investigator, makes the subject ineligible to participate in this study.

### Who will take informed consent? {26a}

The informed consent process will be conducted by the principal investigator or a designated representative. They will thoroughly explain the study's objectives, procedures, and the potential benefits and risks of participation to each prospective patient. Prior to undergoing any study-related procedures or discontinuing any prohibited medication, every patient or their legally acceptable representative must sign and date the informed consent form approved by the Institutional Review Board (IRB).

### Additional consent provisions for collection and use of participant data and biological specimens {26b}

N/a: The collection of all data and biological samples for this study is an integral part of the protocol activities.

## Interventions

### Explanation for the choice of comparators {6b}

In this protocol, one of the groups will receive a placebo, while the other group will receive the P2Et. Both groups will receive chemotherapy treatment and standard care based on routine recommendations for breast cancer. The purpose of including a placebo group is to minimize biases associated with medication administration, particularly in the evaluation and measurement of the desired outcomes. By incorporating a placebo group, the study aims to provide a more rigorous assessment of the effects of the phytotherapeutic intervention. This approach helps to differentiate the specific effects of the phytotherapeutic treatment from any potential placebo effects or other factors related to standard care.

### Intervention description {11a}

A total of 50 patients with breast cancer will be enrolled in this study and randomly assigned to either the treatment group receiving the P2Et extract or the placebo group, with 25 patients in each group. The evaluation will focus on various parameters including immune response, progression-free survival, quality of life, and tumor reduction. Patients in the intervention group will be instructed to take a daily dose of P2Et equivalent to 2000 mg, which is equivalent to 4 capsules. This dose will be divided into two administrations taken with meals, with a time difference of approximately 4 h between each administration. Each administration will consist of 1000 mg (2 capsules) taken every 12 h during the entire duration of the standard therapy.

Patients will self-administer the P2Et phytomedicine according to the prescribed treatment plan until they complete the designated treatment duration. However, they will temporarily suspend the intake of the phytomedicine for a period of 3 days before each cycle of standard therapy, resuming it again 3 days after the completion of each cycle. Specifically, during the anthracycline and cyclophosphamide (AC) phase, patients will begin taking the phytomedicine 3 days after receiving their standard chemotherapy cycle and suspend it 3 days prior to the start of the subsequent cycle. However, for the taxane phase, patients will continue taking the P2Et without any suspension during the treatment cycles.

The P2Et capsules will be manufactured at Labfarve Laboratories under strict adherence to Good Manufacturing Practices (GMP) standards. Each capsule contains 500 mg of dry P2Et extract (DER 20–50:1) extracted using 96% v/v ethanol solvent and additional fractionation processes. The extract is standardized and quantified to contain between 5 to 30% hydrolyzable tannin derivatives calculated as gallic acid (UPLC-UV method), with 2% to 7% consisting of methyl gallate and ethyl gallate.

Quality controls will be conducted in compliance with the regulations set forth by the World Health Organization (WHO), and the Food and Drug Administration (FDA). These quality control procedures will encompass both the active extract and the finished product. Additionally, thorough quality control measures will be implemented to ensure the homogeneity of the final pharmaceutical form for the placebo and the P2Et.

### Criteria for discontinuing or modifying allocated interventions {11b}

Participants have the right to voluntarily discontinue their participation in the study treatment whenever they choose. Furthermore, the investigator has the authority to suspend a participant from the study treatment if necessary due to various reasons, including the occurrence of adverse events or failure to adhere to the study protocol. Instances that may lead to a participant being withdrawn from the study treatment include: the participant or their legally acceptable representative withdrawing their consent, experiencing severe toxicity (grade ≥ 3) when combining P2Et and standard therapy based on NCI CTCAE version 5.0, or if the investigator determines the toxicity to be clinically intolerable. Additionally, suspension may occur if there is a clinically significant elevation that falls below grade 3 or 4, as determined by the investigator. Substantial non-compliance with the protocol that could jeopardize the safety of the participant or compromise the integrity of the data also warrants withdrawal. The investigator may also decide to withdraw a participant if it is believed to be in the participant's best interest. Lastly, a positive pregnancy test will result in immediate withdrawal from the study treatment.

### Strategies to improve adherence to interventions {11c}

All patients receive regular follow-up visits with the principal investigator during their neoadjuvant chemotherapy treatment in each chemo cycle. Each treatment cycle also offers the opportunity for telephone follow-up with patients, giving them the chance to voice any worries or report any symptoms they may have encountered. Furthermore, a dedicated coordinator is responsible for overseeing the administrative aspects of the study, including scheduling follow-up visits, and conducting safety and efficacy assessments throughout the patient's participation in the clinical study. This coordinated approach ensures comprehensive monitoring and support for the patients throughout the study duration.

### Relevant concomitant care permitted or prohibited during the trial {11d}

The neoadjuvant treatment for eligible patients will consist of the AC regimen (doxorubicin/cyclophosphamide) administered every 2 or 3 weeks, followed by either paclitaxel for 12 weeks (4 cycles) or docetaxel every 3 weeks (4 cycles). The specific choice between paclitaxel or docetaxel, as well as the dosing, will be determined by the patient's clinical oncologist based on the national guidelines for stage II-III invasive breast cancer. To manage side effects, antiemetic agents such as setrons and steroids, as well as fosaprepitant, are allowed in this clinical trial. Also, oral setrons, proton pump inhibitors like omeprazole, antidiarrheals such as loperamide (especially with taxanes) may be used. Additionally, colony-stimulating factors such as Pegfilgrastim may be used as needed.

The use of other phytomedicines, vitamins, or herbal supplements should be reported to assess whether they need to be discontinued during the study. The investigator has the discretion to provide appropriate best supportive care and treatment to each subject, which may include interventions such as antibiotics, transfusions, nutritional support, palliative pain management, etc.

### Provisions for post-trial care {30}

All participants will be monitored for a period of 1 month following the completion of their treatment. Additionally, after their final visit, participants will be informed that they have the option to seek clarification or report any new signs or symptoms through telephone or in-person consultations. Furthermore, patients will continue to receive regular clinical follow-up for their underlying disease, adhering to the established schemes and protocols at the Hospital.

### Outcomes {12}

The primary objective of this study is to evaluate the safety and efficacy of P2Et extract in improving the quality of life for breast cancer patients, as well as to explore various treatment outcomes when combined with chemotherapy (Table 1).

**Table 1:** Schedule of activities for the CS003-BC phase II clinical study

Schedule of activities					
**Time point**	**Enrolment**	**Initial Visit and chemotherapy cycle 1 (Anthracyclines)**	**Cycle 2–4 (Anthracyclines)**	**Cycle 5—8 (Taxanes)**	**Close-out**
**Enrolment**					
Screening and Informed Consent	X				
Physical Examen		X	X	X	X
Clinical Record		X	X	X	X
HIV Test and Pregnancy test	X				
**Interventions**					
Medication dispensing		X	X	X	X
Follow-up visit		X	X	X	X
**Safety Assessment**					
Hemogram		X	X	X	X
Transaminase AST		X	X	X	X
Transaminase ALT		X	X	X	X
Alkaline Phosphatase		X	X	X	X
Total Bilirubin		X	X	X	X
Triglycerides		X	X	X	X
Electrocardiogram^a^		X	X		X
Echocardiogram^a^		X	X		X
**Efficacy Assessment**					
RECIST^a^		X	X		X
Metabolome^a^		X	X		X
Microbiome Analysis^a^		X	X		X
Tumor Infiltrate^a^		X	X		X
Progression-Free Survival					X
QLQ C-30 Quality of Life Surveys		X	X		X
QLQ BR-23 Quality of Life Surveys		X	X		X

^a^The mentioned tests will be conducted at three specific time points: before initiating treatment, at the completion of the anthracycline cycle, and at the conclusion of the taxane cycle. Other activities will be performed prior to initiating each cycle or as requested by the researcher for safety assessment purposes

The safety of all participants will be closely monitored by investigators throughout the study. Regular clinical and laboratory assessments will be conducted to identify any evidence of adverse events. Detailed records will be maintained for each adverse event, including the date of onset, diagnosis (if known), signs/symptoms, severity, time course, and the relationship of the event to the study drug. Actions taken in response to the adverse event will also be documented. The National Cancer Institute Common Terminology Criteria for Adverse Events (NCI CTCAE version 5.0) will be utilized to report and grade the severity of adverse events.

The effects on quality of life will be assessed during each patient visit using two validated surveys: the QLQ C-30 version 3 and QLQ BR23. These surveys will be administered prior to each chemotherapy cycle to evaluate the impact on quality of life. The QLQ C-30 version 3 and QLQ BR23 scales have been specifically validated for the Colombian population [22, 23].

To evaluate the objectives related to disease progression, tumor size will be determined through pathological and clinical assessment using radiographic examinations as determined by the treating physician. The Response Evaluation Criteria In Solid Tumors (RECIST 1.1) will be used for analysis, whenever possible.

Disease-free survival will be evaluated in closed cohorts until the evaluation of pathological response. If some patients have not met the necessary criteria at the completion of the protocol, censorship will be applied to account for incomplete data in the survival analysis. Follow-up of these patients will continue until the completion of neoadjuvant treatment or after surgery if they are eligible. Disease-free survival is defined as the time from randomization to disease progression that prevents definitive surgery, local or distant recurrence, development of a second primary malignancy, or death from any cause, whichever occurs first.

### Participant timeline {13}

#### Sample size {14}

The sample size for the study was determined based on the criteria of achieving a difference of at least 10 points in the total score of the quality of life between the two groups, with a standard deviation of 11 points for both groups. With an alpha error of 0.05 (significance level) and a beta error of 0.20 (power of the study), the calculated sample size is 19 patients per group. To account for possible losses during the study, an additional 30% of the sample size was added.

### Recruitment {15}

Patient recruitment for phase II of the study commenced in November 2022 and is anticipated to conclude in October 2023 at the Centro Javeriano de Oncologia of the Hospital Universitario San Ignacio. Preselected patients are contacted and provided with a comprehensive explanation of the study, including the informed consent form. Subsequently, an enrollment visit is conducted to confirm the patient's decision regarding participation. Upon enrollment, each patient is randomly assigned and assigned a dedicated coordinator who will oversee the organization of visits and follow-ups, ensuring smooth execution of study activities. Patients are informed of their right to withdraw from the study at any time without affecting their ongoing clinical treatment.

## Assignment of interventions: allocation

### Sequence generation {16a}

During the screening visit, each participant is assigned a unique subject number in sequential order (e.g., 001, 002, 003, etc.). This assigned number remains the same throughout the study. After the enrollment visit, selected patients undergo randomization using a specially designed application within the electronic case report form (eCRF) in RedCap®. The randomization process follows a simple non-stratified approach, which has been predetermined and loaded into the eCRF for the pharmacist. The pharmacist, who will be unblinded, will carry out the randomization process.

### Concealment mechanism {16b}

The randomization process in this study will be conducted using the electronic case report form (eCRF) module within RedCap®. Prior to the study initiation, the module will be preloaded and customized specifically for this study.

### Implementation {16c}

The recruitment of participants for the study will be carried out by the clinical staff involved in the research. The informed consent process will be conducted by the principal investigator or a designated delegate, following the established procedures at the Hospital Universitario San Ignacio. Once participants have provided their informed consent, the pharmacist will assign them to either the placebo or intervention group for medication dispensing. The blinding of the study will be maintained throughout the duration of the research until all participants have been assigned to their respective groups. The blinding will only be opened by designated personnel responsible for analyzing the results at the conclusion of the study.

## Assignment of interventions: Blinding

### Who will be blinded {17a}

The randomization and treatment assignment process will be managed by the pharmacist, who will be responsible for assigning participants to their respective treatment groups. The pharmacist will have access to the information regarding the treatment assignment of each participant. However, the patients, members of the research team, and investigators will remain blinded and unaware of the treatment assignment throughout the study. This blinding ensures that the study's integrity and objectivity are maintained, as the treatment assignment will not influence the assessment and evaluation of study outcomes.

### Procedure for unblinding if needed {17b}

In the case of serious adverse events requiring unblinding, the pharmacist will request the treatment assignment information and communicate it to the Investigator via email. The unblinding process will be documented in RedCap, the electronic medical record, and officially reported to the Sponsor and Ethics Committee, including the date and reason for unblinding. Monitors with access to unblinded records will ensure adherence to the randomization scheme for quality assurance.

## Data collection and management

### Plans for assessment and collection of outcomes {18a}

Scheduled evaluations outlined in the study protocol will be conducted for all participants, with a dedicated coordinator overseeing the coordination of these activities. Prior to the study, team members will undergo comprehensive training to ensure adherence to protocols and result validity. The administration of scales and evaluations will follow established procedures to maintain consistency and reliability throughout the study.

### Plans to promote participant retention and complete follow-up {18b}

At the beginning of the enrollment process, participants are extensively educated on the importance of following the instructions and recommendations provided by the research team. They are encouraged to voice any questions or concerns and assured of ongoing support through regular phone communication and follow-up visits. If a participant misses a scheduled visit, the research team will take proactive measures to contact their relatives and determine the reason for the absence, ensuring compliance with the planned visits. This proactive approach prioritizes the integrity and comprehensiveness of the study data while emphasizing the well-being and involvement of the participants.

### Data management {19}

For each selected and enrolled subject, an electronic case report form (eCRF) will be completed using a specifically designed electronic data capture system (CED) in RedCap®. The eCRF data are captured by the research center staff, who accurately record all required information specified in the protocol. The subject files maintained by the investigator will serve as the primary source of data for the study and will support the information entered the eCRF. If any necessary corrections need to be made to the eCRF, the investigator or an authorized team member will make the adjustments, and these modifications will be documented in the system's audit trail. Periodic reviews of the eCRFs will be conducted by CRO staff to ensure completeness, legibility, and compliance with the protocol. Access to the source documents will be granted to verify the accuracy of the data entered in the eCRF. The Principal Investigator will review the eCRFs, electronically signing and dating them as confirmation of their review, to ensure completeness and accuracy.

### Confidentiality {27}

The information generated during the clinical study is considered confidential and will be used exclusively for the development of P2Et phytomedicine. This confidential information will be the exclusive property of the sponsors and will not be disclosed to any third parties without written consent. It is strictly prohibited to use this information for any purposes other than the proper execution of the study. Furthermore, the personal data of the participants will be treated as confidential and will not be disclosed during or after the study. Adequate anonymization measures will be implemented to protect the privacy of the participants.

### Plans for collection, laboratory evaluation and storage of biological specimens for genetic or molecular analysis in this trial/future use {33}

N/a: The collection of all data and biological samples for this study is an integral part of the protocol activities. After completing analysis, samples will be discarded following standardized hospital protocol.

## Statistical methods

### Statistical methods for primary and secondary outcomes {20a}

A descriptive analysis of the study database will be conducted to summarize the data. Measures of central tendency (mean, median, and mode) and dispersion (standard deviation and range) will be calculated for quantitative variables, while frequencies and counts will be determined for categorical/ordinal variables. In case of missing data, an active search will be performed using electronic clinical records, laboratory databases, and direct patient inquiry to retrieve the missing information. No imputation method is anticipated, and only patients with complete information will be included in the analysis for each specific objective. For exploratory analyses, the normality of quantitative variables will be assessed to determine the appropriate statistical tests. Parametric tests such as Student's t-test and ANOVA may be used for group comparisons if the data is normally distributed. Non-parametric tests such as the Kruskal–Wallis and Mann–Whitney test will be employed for non-normally distributed data. The Logrank test will be utilized for survival outcomes related to the specified objective.

### Interim analyses {21b}

N/a: No interim analysis is planned for the study until the target sample size is reached.

### Methods for additional analyses (e.g. subgroup analyses) {20b}

N/a: No additional analysis is planned.

### Methods in analysis to handle protocol non-adherence and any statistical methods to handle missing data {20c}

In cases where data is missing, efforts will be made to actively search for the missing information from various sources, including the electronic clinical record system (SAHI®) of the Hospital, the clinical laboratory database (LabCore®), and through direct communication with the patient. However, no imputation method will be used to replace missing data. Only patients with complete information will be included in the analysis for each specific objective.

### Plans to give access to the full protocol, participant level-data and statistical code {31c}

The protocol and the data generated to support the findings of this study will be made available from the corresponding author upon reasonable request.

## Oversight and monitoring

### Composition of the coordinating center and trial steering committee {5d}

All study personnel involved in the research are affiliated with the Hospital Universitario San Ignacio and the Pontificia Universidad Javeriana. They have obtained approval from the ethics committee to participate in the study, ensuring that the research is conducted in accordance with ethical standards. The study will adhere to the principles and guidelines of Good Clinical Practice (GCP), which provide a framework for the design, conduct, monitoring, and reporting of clinical trials. A designated coordinator will be appointed to oversee and coordinate all activities outlined in the study protocol. This coordinator will ensure that all study procedures are followed and that the research is conducted in compliance with the established protocols. Additionally, the research center will have a multidisciplinary team of professionals, including bacteriologists, pharmacist, supporting doctors, and nurses, who have also received approval from the institutional review board (IRB) to actively contribute to the study.

### Composition of the data monitoring committee, its role and reporting structure {21a}

The data monitoring committee, consisting of the leader of the research center's safety research program and an external monitor from the Contract Research Organization (CRO), plays a crucial role in ensuring data integrity. They conduct regular reviews of the electronic case report forms (eCRFs) to assess the quality, legibility, and accuracy of the recorded data. By accessing source documents, they verify the consistency of the data entered in the eCRFs and cross-reference it for validation. Their involvement enhances the overall quality control and oversight of the study, contributing to the reliability and validity of the collected data.

### Adverse event reporting and harms {22}

In this clinical trial, the monitoring and reporting of adverse events (AEs) is a crucial aspect as we are evaluating the safety profile of the P2Et. The investigator will diligently monitor all study participants for any clinical or laboratory evidence of adverse events throughout the study duration. Detailed information about each adverse event will be assessed and recorded, including the date of onset, diagnosis or signs/symptoms, severity, time course, and the relationship of the event to the study medication. If any pregnancies occur, follow-up will be conducted to collect data on the pregnant woman and the newborn. For intermittent adverse events, it is important that they are of a similar nature and severity to be properly recorded. Adverse events will be documented through various means, such as responses to questions, observations by center staff, or self-reporting by the participants themselves. All adverse events will be carefully followed until resolution, either naturally or with appropriate medical intervention, within a maximum period of 4 months from their appearance.

Any adverse events related to the therapeutic intervention of the extract used in the study will be reported. The National Cancer Institute Common Terminology Criteria for Adverse Events version 5 (NCI CTCAE v5) will be utilized for reporting adverse events and grading their severity.

### Frequency and plans for auditing trial conduct {23}

Data will undergo a thorough review as per the monitoring plan to ensure accuracy and maintain quality. The CRO monitor oversees the research center's activities throughout the study. Source documents are reviewed against case report form entries for adherence to protocol and regulations. Discrepancies are cross-checked with written reports and corrected electronically.

### Plans for communicating important protocol amendments to relevant parties (e.g. trial participants, ethical committees) {25}

If any amendments are made to the study, they will be submitted for approval to the ethics committee of the Hospital Universitario San Ignacio. Once approved by the ethics committee, the study will seek approval from the national regulatory agency for implementation. Prior to implementation, comprehensive training will be provided to all personnel involved in the study, tailored to their respective roles and responsibilities within the protocol.

The clinical protocol, informed consents, and investigator's manual used in this study have already been approved by both the ethics committee and the national regulatory agency in their clinical study review. The study will be conducted in accordance with the Good Clinical Practice (GCP) guidelines of the International Conference on Harmonization (ICH), as well as the relevant regulations and standards governing the conduct of clinical studies. Additionally, the study will adhere to the ethical principles outlined in the Declaration of Helsinki.

An amendment was approved by the ethics committee on October 17, 2022 (FM-CIE-0955–22), which included the determination of the optimal biological dose in phase I and the request to open recruitment for phase II.

### Dissemination plans {31a}

The findings from this clinical trial are anticipated to be shared through conference presentations and published in peer-reviewed journals.

## Discussion

The combination of phytomedicines with standard oncological therapy aims to optimize the success of antitumor protocols by improving quality of life, reducing toxic adverse events, increasing response rates, and prolonging progression-free survival. In several of these studies, the herbal preparations are administered alongside standard chemotherapy. These studies primarily evaluate the efficacy, safety, toxicity, adherence feasibility, time to progression, overall survival, quality of life, immunomodulation and pharmacokinetics of different natural preparations and traditional Chinese medicine [24].

One study conducted in breast cancer patients investigated the use of the Japanese herbal medicine TJ-41 (Hochu-ekki-to) to alleviate fatigue during postoperative radiotherapy (RT). In this study, 9 patients with radiation-induced fatigue received TJ-41, and the Functional Assessment Questionnaire for Chronic Disease Therapies-FACIT-F was used to assess outcomes. The results showed that 8 patients (89%) experienced reduced fatigue and improved quality of life [25]. In another study involving 60 patients with advanced non-small cell lung cancer, chemotherapy (gemcitabine and cisplatin) was combined with fermented red ginseng extract. The patients were divided into two groups: one receiving chemotherapy and the extract, and the other receiving chemotherapy alone. The group receiving the ginseng extract showed significant improvements in fatigue symptom scale, Chinese medicine symptom score, psychological status, physical condition, quality of life, and a reduction in chemotherapy toxicity, including leukopenia, thrombocytopenia, nausea, vomiting, and neurological toxicity, compared to the group without the extract [26]. Similar positive effects on quality of life have been observed in patients with advanced-stage non-small cell lung cancer treated with standard therapy using other Chinese herbal medicines [27–29]. In these studies, the polyphenols present in various herbal preparations have shown promising results, particularly in reducing the side effects of chemotherapy and radiotherapy, improving survival rates, and reducing relapses in solid tumors [30, 31].

Despite the existing evidence regarding the potential role of herbal drugs in cancer treatment and their utilization by patients [32], it is essential to conduct clinical studies to thoroughly evaluate their true therapeutic effects. These studies are necessary to establish the efficacy and safety of herbal preparations, as well as to ensure that they do not interact negatively with chemotherapy or other conventional treatments, which could have an impact on the clinical outcomes of patients.

In this phase II study, we aim to assess the safety and explore the efficacy of P2Et, developed following FDA guidelines for herbal drugs [33]. It has met preclinical safety requirements and advanced through phase I trials in healthy volunteers [19, 20] and COVID patients [21]. This contributes to plant-based therapy development, expanding the therapeutic options in breast cancer, enhancing patient quality of life and clinical outcomes.

## Trial status

The recruitment of patients in the phase II started in February 2023 and it is expected to conclude by February 2024 at the Centro Javeriano de Oncologia of the Hospital Universitario San Ignacio if the sample size is achieved.

### Supplementary Information

**Additional file 1. **Supplementary file contains the checklist for the Study Protocols (SPIRIT) 2013 recommendations [34].

## Data Availability

The protocol and the data generated to support the findings of this study will be made available from the corresponding author upon reasonable request.
